# Association between Hypomagnesemia, COVID-19, Respiratory Tract and Lung Disease

**DOI:** 10.2174/1874306402115010043

**Published:** 2021-09-17

**Authors:** Gavino Faa, Luca Saba, Daniela Fanni, Goce Kalcev, Mauro Carta

**Affiliations:** 1 Division of Pathology, Department of Medical Sciences and Public Health, University Hospital San Giovanni di Dio, Cagliari, Italy; 2 Department of Biology, College of Science and Technology, Temple University, Philadelphia, PA, USA; 3 Department of Medical Imaging, Azienda Ospedaliero Universitaria (AOU) of Cagliari-Polo di Monserrato, Cagliari, Italy; 4 International Ph.D. in Innovation Sciences and Technologies, University of Cagliari, Cagliari, Italy; 5 Department of Medical Sciences and Public Health, University of Cagliari, Cagliari, Italy

**Keywords:** COVID-19, Magnesium, Effects, Magnesium deficiency, Lung diseases, Obesity

## Abstract

The complexity of COVID-19 is also related to the multiple molecular pathways triggered by SARS-CoV-2, which is able to cause type I pneumocyte death, trigger intravascular coagulation, interfere with the renin-angiotensin system, dysregulate iron metabolism, ending with the insurgence of a cytokine storm which may lead to death. Old adults with obesity, hypertension, and diabetes are among the high-risk category groups more prone to SARS-CoV-2 infection. Magnesium has been reported to play a major role both in physiology and in pathology, particularly in elderly people, regulating cytotoxic functions of natural killer (NK) cells and CD8+ T lymphocytes. In spite of the absence of controlled trials, the possibility of magnesium supplementation for supportive treatment in patients with COVID-19 should be encouraged. This could be useful in all phases of the COVID-19 disease.

The pandemic Coronavirus disease 2019 (COVID-19), caused by the severe acute respiratory syndrome coronavirus 2 (SARS-CoV-2), is characterized by relevant differences regarding the severity of the disease and the case fatality rate (CFR) across different geographical areas. The high CFR observed in some regions has been hypothesized to be due to multiple factors, such as poor distancing measures, co-morbid conditions, climate [1], pollution [2] health system facilitating access to care [3, 4], genetic characteristics of populations [5, 6] and distribution by the age of populations, with vulnerability for communities with a high percentage of older adults [7]. The complexity of COVID-19 is also related to the multiple molecular pathways triggered by SARS-CoV-2, which is able to cause type I pneumocyte death, trigger intravascular coagulation [8], interfere with the renin-angiotensin system, dysregulate iron metabolism, ending with the insurgence of a cytokine storm which may lead to death [9]. Regarding the co-morbidities, COVID-19 has been hypothesized to may trigger atherosclerotic plaque vulnerability, favoring the invasion of the plaque by inflammatory cells and increasing the risk of developing ischemic stroke and myocardial infarct [10]. The finding that angiotensin-converting enzyme 2 (ACE2), the receptor and the main entry point into human cells for SARS-CoV-2, is a zinc carboxypeptidase, associated with the ability of the virus to dysregulate iron homeostasis with an increase in ferritin serum levels, introduced trace elements among the multiple factors that might explain the marked differences that characterize the clinical course of COVID-19.

Old adults with obesity, hypertension, and diabetes are among the high-risk category groups more prone to SARS-CoV-2 infection, which is often severe or fatal in these subjects [11]. Iron, copper [12], zinc [13], gold [14] and magnesium [15] have been reported to play a major role both in physiology and in pathology, particularly in elderly people. Given that magnesium is an essential trace element involved in over 600 enzymatic reactions in human cells [16] magnesium status might explain, at least partially, why these categories of subjects share an increased risk of severe COVID-19. Hypomagnesemia (serum Mg2+ <0.7 mmol/L) has been described as strongly associated with old age [17] type 2 diabetes mellitus [18] and obesity [19]. Regarding hypertension, hydrochlorothiazide often leads to magnesium deficiency [15]. How could these findings change our knowledge on the linkage between magnesium deficiency and the insurgence of severe pulmonary pathology in patients affected by COVID-19? Recently, it has been hypothesized that a low Mg status might favor the transition from mild to critical clinical manifestations of COVID-19 [20]. Magnesium regulates cytotoxic functions of natural killer (NK) cells and CD8+ T lymphocytes [21]. Decreased NK and T-cell cytotoxicity due to magnesium deficiency may explain the susceptibility of older, hypertensive, obese, and diabetic patients to SARS-CoV-2 infection. In addition, magnesium deficiency upregulates pro-inflammatory cytokine production in monocytes and increases NFkB expression [22]. These data taken together support the role of magnesium deficiency in susceptibility to COVID-19. Moreover, the pro-inflammatory activity of hypomagnesemia substantiates the concept that magnesium deficiency is critically involved in the severe outcome of COVID 19 infections in these categories. Aging, the main determinant of Covid-19 mortality, is often associated with magnesium deficit [23], particularly in poor nutrition conditions such as in non-self-sufficient elderly people living in sheltered homes [24].

Secondary magnesium deficit in aging largely depends on pathologies and treatments common to elderly persons: i.e. non-insulin-dependent diabetes mellitus and use of diuretics [25]. Thus, several conditions occurring in old adults are recognized as risk factors for Covid mortality (i.e. vascular diseases [26, 27] are well known associated with magnesium deficit. More specifically, the presence of clinical and biochemical correlates of low serum and muscle Mg was found by means of muscle biopsy and blood samples in people consecutively admitted to a pulmonary Intensive Care Unit for chronic obstructive pulmonary disease and acute respiratory failure [28]. This may also suggest a short circuit in Covid respiratory conditions in which the deficit supported by the respiratory impairment may lead to worsening of infection. Although we do not believe that controlling magnesium in the elderly may be the key to solving the Covid, however, these considerations lead us to think that greater attention should be paid to this issue.

## CONCLUSION

To conclude, there are several reasons to believe that magnesium deficiency may predispose to COVID-19, ending with severe pulmonary disease, often fatal. What are, in summary, the major links between magnesium deficiency and severe COVID-19? A recent Editorial by the president of the German society for magnesium research lays stress on the role of magnesium in the regulation of antiviral immunity and in the prevention of QT interval prolongation, possibly caused by multiple drugs for the treatment of COVID-19 [29]. More than 30% of COVID-19 patients with QT prolongation show hypomagnesemia [30]. Keeping serum Mg levels above 3 mg/dL in COVID-19 patients was effective in preventing QT-prolonging and sudden cardiac arrest [31]. Hypomagnesaemia due to the use of proton-pump inhibitors has been linked to a worse outcome in patients with COVID-19 [32].

ATP regeneration following the cytokine storm induced by SARS-CoV-2 requires adequate magnesium levels, and is deficient in patients with hypomagnesemia, causing a more severe form of COVID-19 [33]. Hypomagnesemia in COVID-19 patients may cause the calcium “channel blocking effects” played by magnesium ions in physiology, which lead to suppression of interleukin-6 and other endocrine disruptors, leading to the exaggerated inflammatory response typical of more severe COVID-19 patients [34]. A combined oral treatment of older COVID-19 patients with Magnesium, vitamin D and vitamin B12 reduces the proportion of patients requiring oxygen and intensive care support [35].

May these findings change our clinical practice? Data mainly suggests that hypomagnesemia is an issue, so magnesium supplementation may help in a patient who may be magnesium deficient and may not be helpful in patients who have normal magnesium levels. According to these data, a subset of COVID-19 patients with hypomagnesemia may really benefit from magnesium supplementation [36]. Importantly, magnesium supplementation may restore intracellular magnesium, with the recovery of NK cells and CD8 lymphocytes and decreased cytokine production [21]. In spite of the absence of controlled trials, the possibility of magnesium supplementation for supportive treatment in patients with COVID-19 [36-38] should be encouraged. This could be useful in all phases of the disease [39] and adequate studies should be conducted on this important aspect both on the therapeutic and preventive side. In fact, this problem can be common to many respiratory conditions as well as COVID-19 infection.
